# Innovative Fly Ash Geopolymer-Epoxy Composites: Preparation, Microstructure and Mechanical Properties

**DOI:** 10.3390/ma9060461

**Published:** 2016-06-09

**Authors:** Giuseppina Roviello, Laura Ricciotti, Oreste Tarallo, Claudio Ferone, Francesco Colangelo, Valentina Roviello, Raffaele Cioffi

**Affiliations:** 1 INSTM Research Group Napoli Parthenope, Centro Direzionale Napoli, Dipartimento di Ingegneria, Università di Napoli ‘Parthenope’, Isola C4, Napoli 80143, Italy; giuseppina.roviello@uniparthenope.it (G.R.); claudio.ferone@uniparthenope.it (C.F.); francesco.colangelo@uniparthenope.it (F.C.); raffaele.cioffi@uniparthenope.it (R.C.); 2 Dipartimento di Scienze Chimiche, Università degli Studi di Napoli “Federico II”, Complesso Universitario di Monte S. Angelo, via Cintia, Napoli 80126, Italy; oreste.tarallo@unina.it; 3 Dipartimento di Ingegneria Chimica, dei Materiali e della Produzione Industriale, p.le Tecchio 80, Napoli 80126, Italy; valentina.roviello@unina.it

**Keywords:** geopolymer, fly ash, composites, epoxy resin, SEM, mechanical properties

## Abstract

The preparation and characterization of composite materials based on geopolymers obtained from fly ash and epoxy resins are reported for the first time. These materials have been prepared through a synthetic method based on the concurrent reticulation of the organic and inorganic components that allows the formation of hydrogen bonding between the phases, ensuring a very high compatibility between them. These new composites show significantly improved mechanical properties if compared to neat geopolymers with the same composition and comparable performances in respect to analogous geopolymer-based composites obtained starting from more expensive raw material such as metakaolin. The positive combination of an easy synthetic approach with the use of industrial by-products has allowed producing novel low cost aluminosilicate binders that, thanks to their thixotropicity and good adhesion against materials commonly used in building constructions, could be used within the field of sustainable building.

## 1. Introduction

Geopolymers are a family of inorganic materials obtained by reaction between an aqueous alkaline silicate solution and an aluminosilicate source [1,2]. This reaction yields an amorphous three-dimensional structure in which SiO_4_ and AlO_4_ tetrahedra are linked by corner-shared O atoms.

Geopolymers are characterized by interesting mechanical properties, low shrinkage, thermal stability, freeze-thaw, chemical and fire resistance, long term durability and recyclability. For these reasons, they have the potential for utilization as Ordinary Portland Cement (OPC) replacement in a wide range of applications, such as fireproof barriers, materials for high temperatures, matrices for hazardous waste stabilization, toolings and moldings [3,4].

Moreover, with respect to the manufacturing of OPC that consumes a significant amount of natural materials and energy release of a large quantity of carbon dioxide in the atmosphere [5,6,7,8], the use of geopolymer-based materials in concrete applications could significantly reduce the CO_2_ emissions [9] thanks to the “low carbon” footprint of several raw materials with a high concentration of aluminosilicates from which they can be prepared, *i.e.*, dehydroxylated kaolinite (metakaolin, MK) or industrial waste such as fly ash.

Fly ash (FA) is a fine powder by-product transported by flue gas after the combustion of coal in coal-fired power stations typically made up of small glass spheres, consisting primarily of silicon, aluminium, iron, and calcium oxides [10,11,12,13,14]. The development of FA-based geopolymer concretes could contribute to recycling waste into construction material, thus reducing, at the same time, CO_2_ emissions [15,16,17].

**Figure 1:**
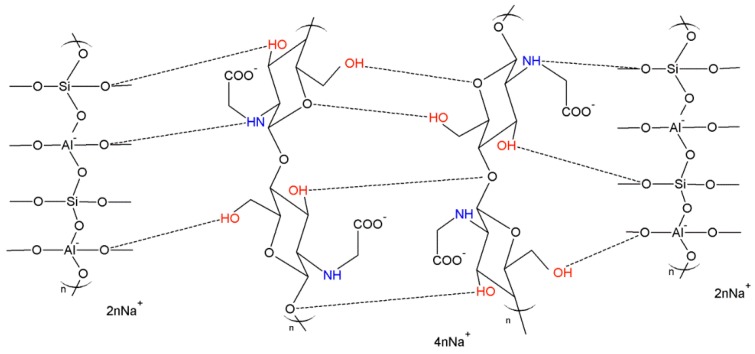
Schematic representation of the mechanism of interaction between geopolymer (on the left and on the right, in the hypothesis of Na/Al = 1:1) and *N*-carboxymethyl chitosan macromolecules (in the middle). Dashed lines represent hydrogen bonds [18].

However, FA-based geopolymers typically exhibit brittle behavior with low tensile strength, ductility, and fracture toughness, thus limiting up to now the actual possibility to use these very promising materials for extensive and practical applications in constructions. This limit could be, in principle, overcome by developing geopolymer composites, but, to the best of our knowledge, very little is reported on this topic. Only recently, Li *et al.* [18] described the utilization of chitosan biopolymers for the implementation of composite systems based on geopolymers obtained from fly ash, in which the formation of a three-dimensional cross-linked composite is achieved by means of a network of hydrogen bonds between geopolymer and chitosan macromolecules (Figure 1).

In that case, the interaction between the inorganic matrix and the macromolecule is effective up to 0.1% by weight of *N*-carboxymethyl chitosan content, while a further increase of the chitosan amount produces a decrease of the geopolymerization degree, probably due to an encapsulation effect of the fly ash particles that become un-reactive [18]. It is worth noting that, in a bid to obtain advanced geopolymer-based composite materials, a wide range of organic polymers have been studied in literature as organic fillers, such as polyvinyl acetate [19], polypropylene [20], polyvinyl alcohol [21], or water-soluble organic polymers [22]. These kind of composites are usually obtained by blending the polymer with geopolymers, sometimes in the presence of compatibilizers [23,24,25]. Nevertheless, a worsening effect of the mechanical properties of the final product [26] for very low concentrations (up to ~1 wt %), similar to that previously described in the case of chitosan, has been observed. In these last cases, this detrimental effect is probably due to the use of organic materials poorly compatible with the inorganic matrix that, at high amounts of the organic components, causes a phase separation between the organic filler and the inorganic geopolymer matrix.

**Figure 2:**
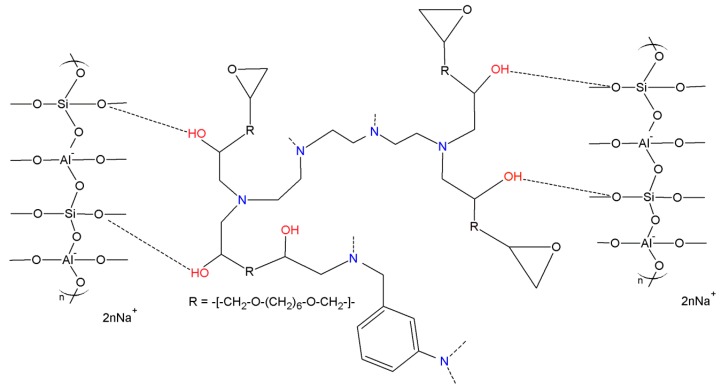
Schematic representation of some possible interactions between the epoxy resin Epojet^®^ [31] (in the middle) and the geopolymeric matrix (on the left and the right, in the hypothesis of Na/Al = 1:1). Dashed lines represent hydrogen bonds.

Very recently, we have developed an innovative, easy and cost-effective synthetic strategy to realize hybrid composite materials by using metakaolin-based geopolymers as inorganic components and different organic resins up to 25% in weight [27,28,29,30,31,32]. These materials show significantly improved physical and mechanical properties and remarkably reduced brittleness with respect to the neat geopolymers, still preserving good thermal and fire resistance [30,32]. This approach consists of the concurrent co-reticulation of both phases that are mixed together when each polymerization reaction is already started but is far from being completed, thus allowing for realization of a chemical interaction between the organic component and the geopolymeric mixture based on the formation of a wide network of hydrogen bonding (Figure 2) [29]. This approach ensures high compatibility between the phases and a very good dispersion even at the nanometric level of the organic phase that can be included in the mixture up to about 25% by weight, without addition of external additives or compatibilizers [30].

In the present paper, we report on the preparation of novel FA-based geopolymer composites, obtained by extending the synthetic method developed by us in the case of more expensive metakaolin-based geopolymers to raw waste materials. In such a way, we have succeeded in combining an easy and sustainable synthetic approach with the use of industrial by-products for the realization of novel “eco-friendly” aluminosilicate binders in order to preserve the environment and limit the cost of construction materials.

These new promising materials have been characterized by means of several techniques, such as scanning electron microscopy (SEM), thermal analysis (TGA), X-ray diffraction (XRD), and compressive strength tests. Their properties have been compared with neat geopolymer samples and analogous samples obtained from metakaolin [28].

## 2. Experimental Section

### 2.1. Materials and Methods

Class F coal fly ash employed in this work was supplied by the ENEL S.p.A. power plant located in Brindisi (Southern Italy) and was used as received, without drying treatment. The water content was found to be 4% after drying in an oven at 105 °C until reaching a constant mass. Its chemical composition was obtained by means of a Perkin-Elmer Optima 2100 DV ICP-OES apparatus (Waltham, MA, USA) [14,33] and is reported in Table 1. The water content of fly ash was taken into account in the mix design. Metakaolin was kindly provided by Neuchem S.r.l. (Milan, Italy), and its composition is reported in Table 1. Epojet^®^ epoxy resin [34] was purchased by Mapei S.p.A. (Milan, Italy). Sodium hydroxide was purchased from Sigma Aldrich (St. Louis, MO, USA) and used without further purification. The sodium silicate solution was supplied by Prochin Italia S.r.l. (Naples, Italy), and its composition is reported in Table 1.

The fly ash particle size distribution was determined by means of a Malvern Mastersizer 3000 laser particle analyser (Malvern, UK). Thermogravimetric analyses (TGA) were performed by a Mettler-Toledo TGA/DSC2 STAR^e^ SYSTEM (Columbus, OH, USA). The thermographs were obtained with a heating rate of 10 °C/min using ≈10 mg of the powdered sample under air flow. X-ray diffraction patterns were obtained at room temperature with an automatic Rigaku powder diffractometer mod. Miniflex 600 (Tokyo, Japan), operating in the θ/2θ Bragg-Brentano geometry. The phase recognition was carried out by using the PDF-4+ 2014 (International Centre for Diffraction Data^®^, Tokyo, Japan) database and the Rigaku PDXL2 software (Rigaky, Tokyo, Japan). SEM analysis was carried out by means of a Nova NanoSem 450 FEI Microscope (Hillsboro, OR, USA). The compressive strength was evaluated according to EN 196-1 and measured by testing cubic paste specimens (30 × 30 × 30 mm^3^) in a Controls MCC8 multipurpose testing machine (CONTROLS s.r.l., Liscate, Milan, Italy) with a capacity of 100 kN. The tests were performed after 28 days of curing at room temperature, and the values reported are the averages of the five compression strength values.

### 2.2. Specimen Preparation

#### 2.2.1. Preparation of Metakaolin-Based Geopolymer (G-MK)

The alkaline activating solution was prepared by dissolving solid sodium hydroxide into the sodium silicate solution. The solution was then allowed to equilibrate and cool for 24 h. The composition of the obtained solution can be expressed as Na_2_O·1.34SiO_2_ 10.5H_2_O. Then, the metakaolin was incorporated to the activating solution with a liquid to solid ratio of 1.4:1 by weight and mixed by a mechanical mixer for 10 min at 800 rpm. The composition of the whole geopolymeric system can be expressed as Al_2_O_3_ 3.5SiO_2_ 1.0Na_2_O·10.5H_2_O, assuming that geopolymerization occurred at 100%. Such composition is in good agreement with that actually determined by EDS analyses on the prepared samples.

#### 2.2.2. Preparation of Fly Ash-Based Geopolymer (G-FA)

The alkaline activating solution was prepared by mixing the sodium silicate solution with an aqueous solution of sodium hydroxide 10 M. The solution was then allowed to equilibrate and cool for 24 h. The composition of the obtained solution can be expressed as Na_2_O 0.9SiO_2_ 14.7H_2_O. Then, fly ash was incorporated into the activating solution with a liquid to solid ratio of 0.66:1 by weight and mixed by a mechanical mixer for 20 min at 800 rpm. The composition of the whole geopolymeric system can be expressed as Al_2_O_3_ 3.79SiO_2_ 0.66Na_2_O 8.9H_2_O, assuming that geopolymerization occurred at 100% (see the related discussion in Section 3.1.4).

#### 2.2.3. Preparation of the EPOXY-Geopolymer Composites

Geopolymer-based composites have been obtained by adding Epojet^®^ resin to the freshly-prepared geopolymeric suspension, and were quickly incorporated by controlled mixing (5 min at 1350 rpm). Epojet^®^ is a commercial two-component epoxy adhesive for injection, which, after mixing, takes on the qualities of a low viscosity liquid. It is obtained by mixing its components in 4:1 ratio in weight, as specified in the technical data sheet, supplied by the manufacturer [34], and it is usable for 40 min at room temperature. Before being added to the geopolymeric mixture, Epojet^®^ was cured at room temperature for 10 min. The resin was added when it was still easily workable and long before its complete crosslinking and hardening (that takes place in about 5–7 h at 23 °C). It is worth noting that the addition of the unreacted components of the resins to the inorganic suspension produces phase segregation and, on the contrary, a late mixing of the two components (the cured organic resin and the geopolymer) results in a strongly reduced homogeneity of the final material.

Different specimens containing up to 20% *w*/*w* of resin were prepared: in particular, G-FA-Ep10 and G-FA-Ep20 specimens were obtained by mixing Epojet^®^ epoxy resin (10% and 20% by weight, respectively) with fly ash-based geopolymer (G-FA); G-MK-Ep10 and G-MK-Ep20 were obtained by mixing Epojet^®^ epoxy resin (10% and 20% by weight, respectively) with metakaolin-based geopolymer (G-MK). All the composites started solidifying in few minutes [28].

The composition of the studied samples is reported in Table 2.

### 2.3. Curing Treatments

As soon as prepared, MK-based specimens were casted in cubic molds and cured at room temperature (=25 °C) in >95% relative humidity conditions for seven days. The evaporation of water was prevented by sealing the top of the molds with a thin plastic film during the curing stage. The specimens were left for a further 21 days in air at room temperature before being characterized.

FA-based specimens, as soon as prepared, were casted into cubic molds and cured at 60 °C for 48 h in >95% relative humidity conditions (the evaporation of water was prevented by sealing the top of the molds with a thin plastic film during the curing stage) and further five days in >95% relative humidity conditions at room temperature. Finally, the specimens were left for a further 21 days in air at room temperature before being characterized.

## 3. Results and Discussion

### 3.1. Characterization

#### 3.1.1. X-ray Diffraction Characterization

**Figure 3:**
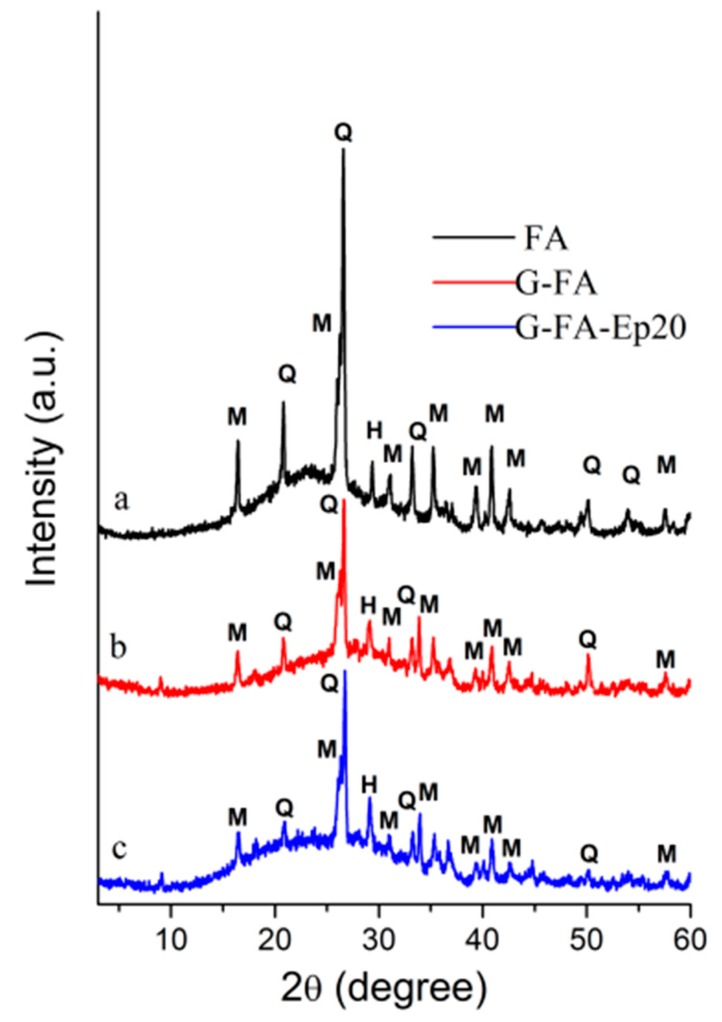
X-ray powder diffraction patterns of (**a**) the fly ash (FA; **black** line); (**b**) the cured neat geopolymer (G-FA; **red** line); and (**c**) the composite sample (G-FA-Ep20; **blue** line). H = hematite; M = mullite; Q = quartz.

Figure 3 shows the XRD patterns of the used fly ash (FA), the cured neat FA-based geopolymer (G-FA), and the composite sample (G-FA-Ep20) containing 20 wt % of organic resin. The diffraction pattern of the fly ash is characterized by a wide and diffused hump in the interval range 15°–35° 2θ with a maximum at 2θ°–25°. Minor crystalline phases such as quartz (JCPDS 01-070-2517), mullite (JCPDS 01-076-2579) and hematite (JCPDS 00-013-0534) may also be identified. This amorphous halo is shifted towards slightly higher angular values (maximum at 2θ°–30°) in the G-FA sample, indicating the formation of an alkaline aluminosilicate hydrate gel (N-A-S-H) with a 3D amorphous structure [35]. The crystalline phases detected in the initial material (quartz, mullite and hematite) remain substantially unaltered. In this respect, it is known that, in the fly ash only, the amorphous aluminosilicate component is reactive in the geopolymerization reaction [36]. Finally, as far as the composite specimen is concerned, the X-ray diffraction pattern is very similar to that of the FA-based geopolymer, even if it is characterized by a more pronounced amorphous halo due to the presence of the organic resin.

#### 3.1.2. Thermal Analysis

**Figure 4:**
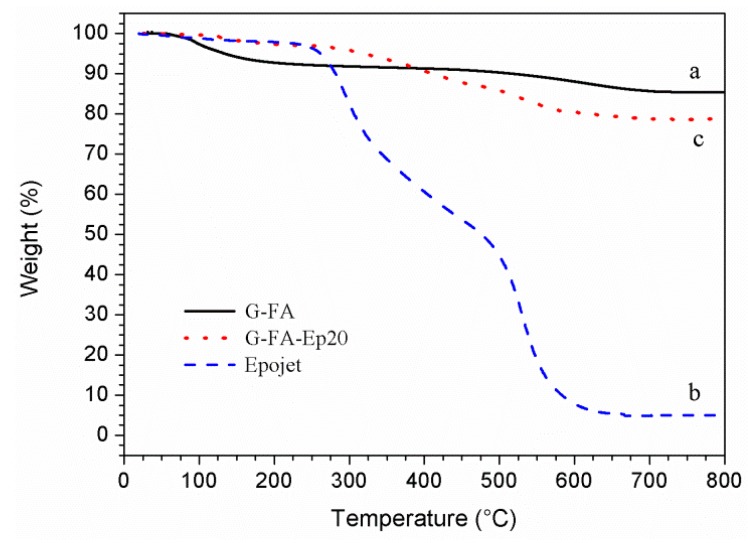
TGA curves of (**a**) the cured G-FA neat geopolymer sample (**black** solid line); (**b**) the pure epoxy resins (Epojet, **blue** dashed line); and (**c**) the G-FA-Ep20 composite specimen (**red** dotted line).

Thermogravimetric analysis was performed on the neat G-FA geopolymer, on the organic resin, and on the G-FA-Ep20 composite (Figure 4) after the curing.

In the case of the neat G-FA geopolymer specimen, weight loss starts at =30 °C and is completed at 750°. The overall weight loss is 15% and can be attributed to the removal of water molecules absorbed (up to ≈100 °C) or differently linked (up to ≈200 °C, free water in the pores; at higher temperatures, structural water and bound water in the nanopores) to the silicate molecules [37,38,39].

Epojet^®^ resin shows a degradation mechanism involving two main steps. The resin is thermally stable up to about 250 °C. Above this temperature, a first degradation step that finishes at ≈480 °C is observed, resulting in a weight loss of 51%. The second degradation process is completed at about 650 °C and a combustion residual of about 5% remains.

As far as the G-FA-Ep20 composite specimen, the weight loss shows a complex mechanism involving different steps: in particular, a first step, corresponding to a weight loss of ≈4%, is recorded up to ≈300 °C, while a second step, characterized by a complex path, is observed from ≈300 °C up to ≈700 °C and corresponds to a further weight loss equal to ≈22%. It was found [28] that the first degradation step is associated mainly with the loss of water of the geopolymeric phase while the second one corresponds to the degradation of the dispersed organic phase. The combustion residual at 800 °C is about 78%. Moreover, it is worth pointing out that, from the DTA of the curves reported in Figure 4, the peak temperature of water loss for the composite (130 °C) is higher than that of the pure geopolymer (95 °C): probably, the polar groups of the resin interact with the water molecules, delaying their evaporation [28] (see column numbers 4 and 5 of Table 3).

Degradation temperatures and weight losses for the studied systems are summarized in Table 3.

#### 3.1.3. Microstructural Analysis of Fly Ash

**Figure 5:**
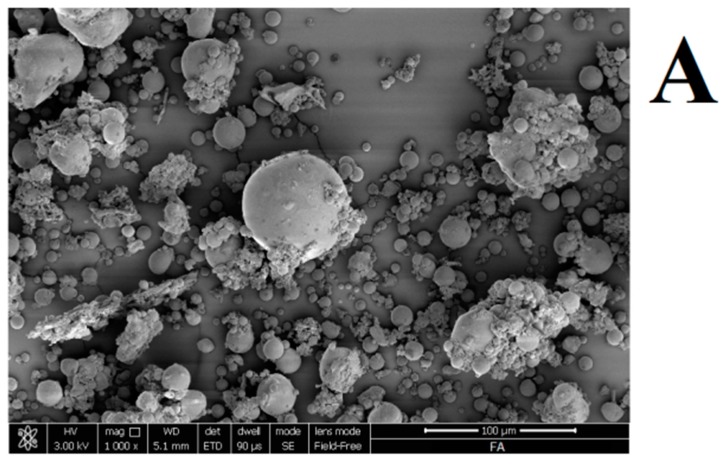

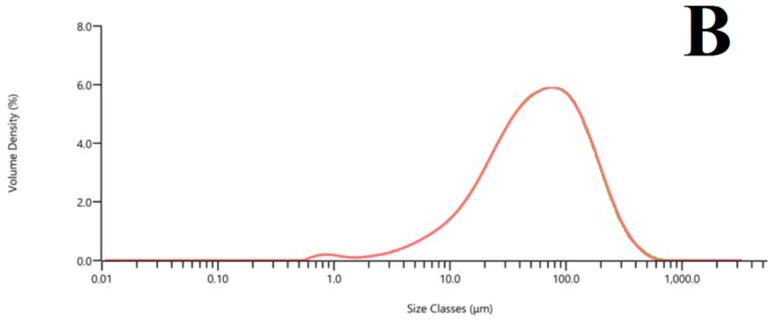
(**A**) scanning electron microscope (SEM) micrographs and (**B**) particle size distribution (volume density *vs.* size) of the used fly ash.

Figure 5 shows SEM micrographs of the used fly ash and their particle distribution. It is apparent that the fly ash consists mostly of glassy cenospheres. Such microstructure is in good agreement with that reported in the literature [2,12,13,14,15]. Moreover, the dimensions of the particles and their aggregates range between a few microns to ≈30 microns have been detected. In particular, the D_50_ is 30.1 μm (Figure 5B).

#### 3.1.4. Microstructural Analysis of Geopolymers and Geopolymer Based Composites

**Figure 6:**
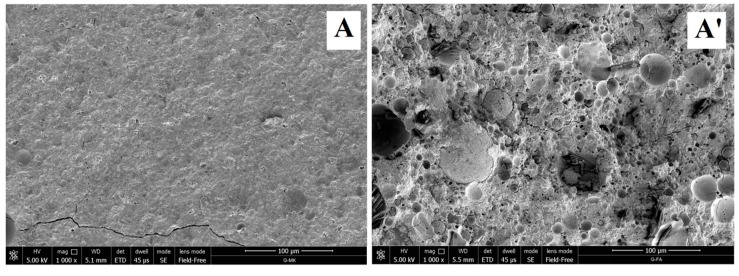

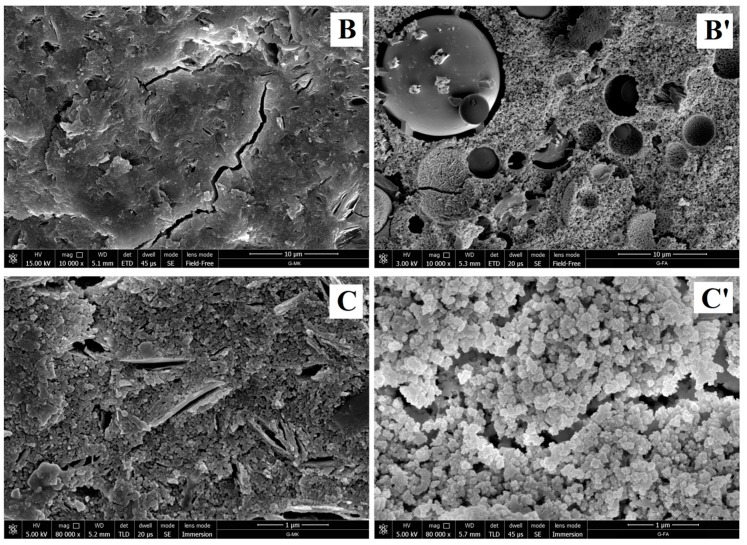
Scanning electron microscope (SEM) micrographs of MK-based geopolymer (**A**–**C**) and FA-based geopolymer (**A’**–**C’**).

The SEM micrographs of freshly obtained fracture surfaces of the MK-based geopolymer and FA-based geopolymer are reported in Figure 6.

MK based-geopolymer (Figure 6A–C) is characterized by a compact morphology that only at very high magnification (Figure 6C) reveals some unreacted kaolinite crystals and the presence of small spheroidal domains, probably reminiscent of the gelation process of the geopolymer.

The morphology of FA-based geopolymer (Figure 6A’–C’) instead is dominated by the presence of the unreacted FA particles that are well dispersed in the geopolymer matrix. This disaggregated morphology is typical of fly ash-based geopolymers [1,33,40] Moreover, in this sample, the globular morphology of the geopolymeric matrix is rather evident already at 10,000 magnification (Figure 6B’) and, at variance with the MK-based geopolymer for which the particle dimensions are in the range 20–50 nm (Figure 6C), in this last case, matrix globules have bigger average diameters, in the range 50–100 nm (Figure 6C’).

The limited reactivity of the fly ash particles causing the non-completeness of the geopolymerization reaction also influences the composition of the geopolymer matrix that, as determined by EDS analysis performed on a homogeneous moiety of the surface, is characterized by Si:Al and Na:Al ratios equal to 1.11 and 0.16, respectively.

**Figure 7:**
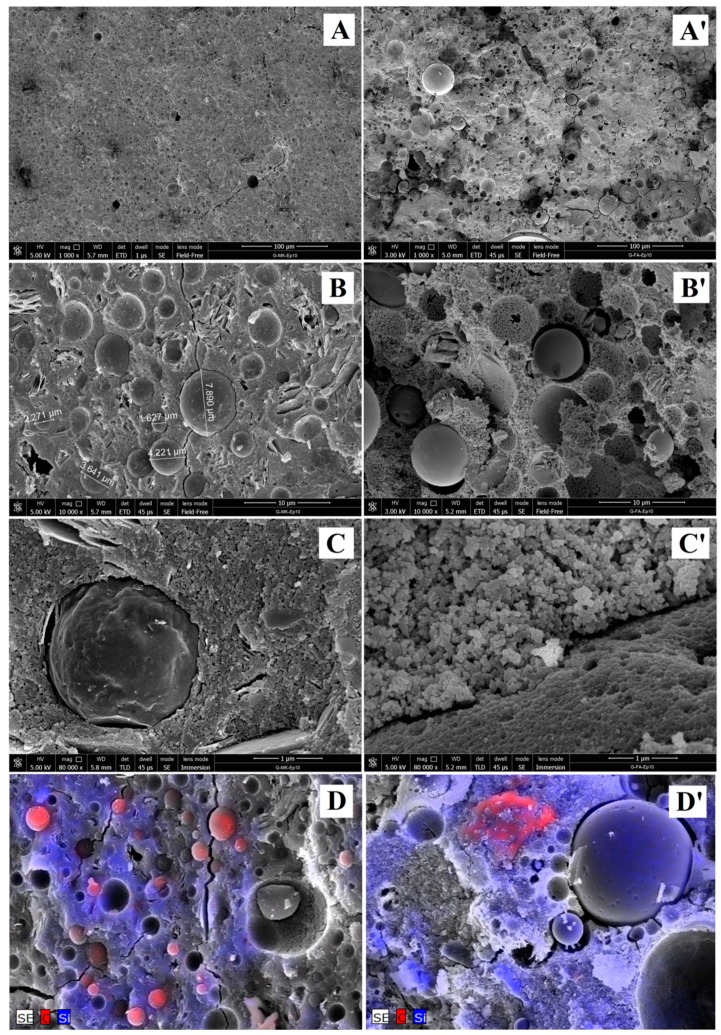
Scanning electron microscope (SEM) micrographs of **MK**-based geopolymer composites (G-MK-Ep10; (**A**–**C**)) and FA-based geopolymer composites (G-FA-Ep10; (**A’**–**C’**)). In (**D**,**D’**), the **EDS** maps (at 5000 magnification) of silicon (in **blue**) and carbon (in **red**) of two representative regions of the G-MK-Ep10 and the G-FA-Ep10 sample are shown, respectively.

In Figure 7, the SEM micrographs of the FA-based geopolymer composite containing the epoxy resin (10% by weight) are reported (A’–C’) and compared to the images of the MK-based samples having the same resin content (A–C).

As far as the MK-based sample, resin particles are homogeneously dispersed in the geopolymer matrix as well-defined microspheres with diameters in the range 1–10 μm. No segregation phenomena are observed (Figure 7A,B) and a good adhesion between the organic phase (resin) and the inorganic one (geopolymer matrix) is apparent (Figure 7C) [28].

In the case of the FA geopolymer composite, particles of organic resin strongly interacting with the matrix are still observed (Figure 7C’) but, in respect to the MK-based composite sample, an increase of the dimensions and a less homogeneous distribution of the particles is observed, probably due to the lower reactivity of FA in respect to MK, that allows the coalescence of the drops of resin during the geopolymerization reaction. In addition, several microspheres of unreacted fly ash particles are still present. These particles could act as a reinforcing agent of the matrix, improving the mechanical properties with respect to the neat geopolymer (see Section 3.1.5). In order to help distinguish between these two types of particles (*i.e.*, the organic resin particles containing carbon and the unreacted fly ash particles), Figure 7D,D’ shows the EDS maps of the elements carbon (in red) and silicon (in blue) for the G-MK-Ep10 and G-FA-Ep10 composites. It is worth noting that, in the case of G-MK-Ep10, it is evident that the organic particles are scratched when the samples are broken to prepare the SEM specimens (Figure 7B,C), while the unreacted fly ash spheroidal particles preserve their very smooth surface (Figure 7B’).

**Figure 8:**
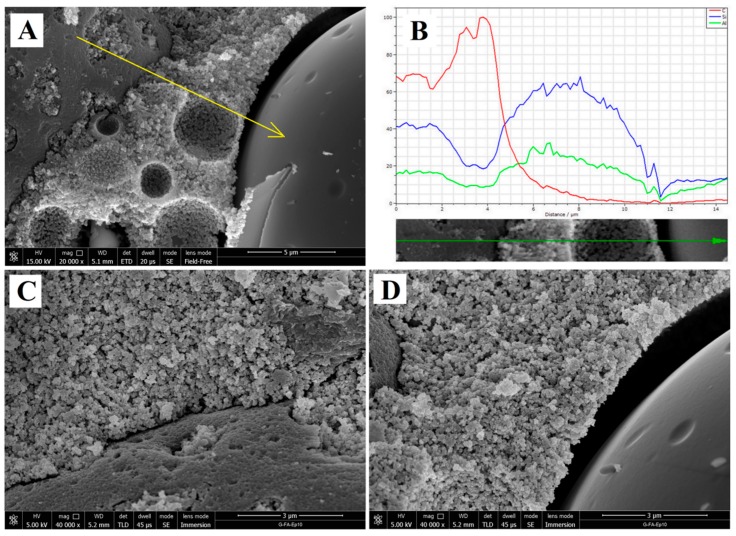
SEM image of the sample G-FA-Ep10: (**A**): magnification at 20,000× of a part of Figure 6B’ that shows, following the direction of the yellow arrow: (i) the resin particle; (ii) the matrix and (iii) the not-reacted ash particle; (**B**) % distribution of C (**red**), Si (**blue**), Al (**green**) along the arrow obtained by EDS analyses; (**C**,**D**) 40,000× magnification image of the interface zone between the geopolymer matrix and a resin particle, and between the matrix and an ash particle, respectively.

These experimental pieces of evidence reflect the different interactions of the resin and FA particles with the geopolymeric matrix, as also shown in Figure 8. In particular, the interaction of the resin particles with the inorganic matrix is very strong due to the network of hydrogen bonds between the phases (Figure 7C’ and Figure 8C) [28]. On the contrary, there is a clear gap between unreacted FA particles and geopolymer matrix (Figure 8D).

Figure 8B reports the EDS analysis taken along a line starting on a resin particle, passing through the geopolymer matrix and coming to an FA particle (the arrow is shown in Figure 8A in yellow). By recording the variation of the chemical composition of carbon, silicon and aluminium along this line, it is apparent that the resin particle is characterized as expected by a high content of carbon and by the presence of minor Si and Al due to the adhesion of geopolymer matrix on its surface. Both geopolymeric matrix and fly ash particle instead show a negligible content of C while Si and Al are present. In particular, in agreement with the chemical composition (see Paragraph 2.1), FA particles are characterized by a similar content of Si and Al, while, in the geopolymer matrix, the silicon content is higher than that of aluminium.

#### 3.1.5. Compressive Strength Test

**Figure 9:**
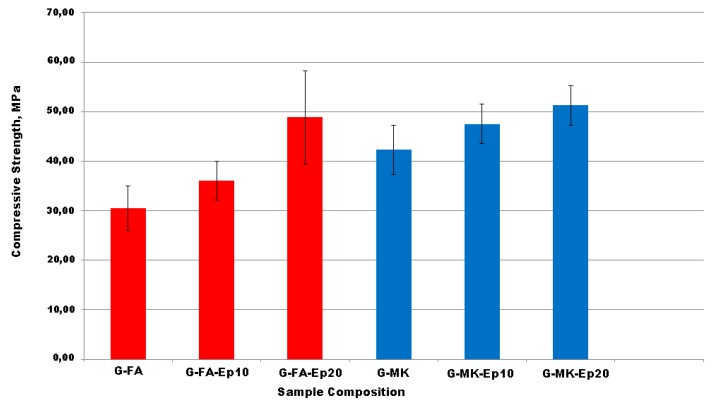
Compressive strength (MPa) of G-FA, G-FA-Ep10, G-FA-Ep20 (in **red**) and G-MK, G-MK-Ep10, G-MK-Ep20 (in **blue**).

The compressive strengths of FA-based geopolymers (G-FA) and of the corresponding composites containing, respectively, 10% and 20% by weight of epoxy resin (G-FA-Ep10, G-FA-Ep20) are reported in Figure 9 and compared with the mechanical performances of the composite specimens obtained starting from metakaolin and containing the same resin content (G-MK, G-MK-Ep10, G-MK-Ep20).

As expected on the basis of the limited reactivity of the weathered fly-ash with respect to MK-based geopolymers [14,33], G-FA samples show a lower compressive strength than G-MK.

In particular, by comparing the values of mechanical strength (Figure 9), it is possible to observe that, in the case of neat geopolymer samples, G-MK mixture is characterized by a noticeable increase of ≈40% of compressive strength in respect to G-FA mixture.

As far as composite samples, for both the matrices, the incorporation of the organic resin in the neat geopolymeric material significantly influences their mechanical properties, as the compressive strength increases with the organic content (compare G-FA-Ep10 and G-FA-Ep20 *vs.* G-FA and G-MK-Ep10 and G-MK-Ep20 *vs.* G-MK) and, in both cases, the best mechanical performance was obtained for the specimen containing 20% by weight of organic resin. As already demonstrated for the metakaolin composites, this evident improvement of mechanical properties is probably due to the presence of the organic resin that acts as reinforcement, thanks to a crack deviation mechanism and absorbing part of the load by plastic deformation [28].

Moreover, it is worth noting that the differences in the mechanical performances of FA-based composites and MK-based ones strongly decrease as the organic resin content increases. In particular, the difference in compressive strength of composite samples is ≈30% for the samples containing 10% by weight of resin (G-MK-Ep10 and G-FA-Ep10) and is ≈5% only in the case of the samples containing 20% by weight of resin (G-MK-Ep20 and G-FA-Ep20).

These data allow for concluding that the presence of the resin is more effective when it is used with the fly ash-based geopolymer samples, rather than the metakaolin ones.

This observation supports the idea of a valid use of fly ash in place of more expensive raw materials, such as metakaolin, in particular for those applications for which it is important to save materials and limit the costs.

Finally, preliminary data (not reported) indicate that, if compared to neat G-FA, G-FA composite mixtures show an improved adhesion to common construction supports, minimizing pouring phenomena and avoiding aggregate segregation.

## 4. Conclusions

Through a co-reticulation reaction of commercial epoxy-based organic resins and a fly ash-based geopolymer, new organic–inorganic composite materials with improved mechanical properties were prepared.

This strategy allows the organic phase to chemically interact with the geopolymeric mixture during the geopolymerization process through the formation of a wide network of hydrogen bonding due to the presence of several hydroxyl groups. In such a way, a high compatibility between the organic and inorganic phases, even at appreciable concentration of resin (20% *w*/*w*), was realized up to micrometric level and a good and homogeneous dispersion (without the formation of agglomerates) of the organic particles was achieved.

These new materials show enhanced mechanical properties in respect to the neat geopolymer. In particular, compressive strength of the new composite material is significantly higher than that of the neat fly-ash based geopolymer, being comparable or even superior to that obtained starting from the more expensive metakaolin.

It is worth pointing out that, despite the high concentration of organic resin, similarly to the analogous composites containing melamine based resins [30], preliminary data show that these new materials are not flammable and do not produce smoke in significant amounts.

Considering the increasing demand for materials with low environmental impact in today’s construction and housing industry, this paper tries to add new results in the field of sustainable building materials with reduced environmental footprint. In fact, the following conclusions can be drawn:

1. the compressive strength of the composite materials is significantly better than that of the neat geopolymer;
2. fly ash-based composite materials represent a valid alternative in place of more expensive raw materials, such as metakaolin, in particular for those applications for which it is important to save materials and limit the costs;
3. the procedure is inexpensive and uses easily available reagents.

Finally, having successfully replaced the metakaolin with fly ash, we can suggest that the novel composites may have all the conditions to be an Environmentally Friendly Material. In order to confirm this hypothesis, a complete LCA (Life Cycle Assessment) study is in progress.

## Figures and Tables

**Table 1:** Chemical composition (weight%) of weathered fly ash used in this paper after thermal treatment at 950 °C, metakaolin and the sodium silicate solution.

**Fly Ash**							
Al_2_O_3_	SiO_2_	K_2_O	Fe_2_O_3_	Na_2_O	MgO	CaO	others
28.12	53.75	1.89	6.99	0.87	1.59	4.32	2.47
**Metakaolin**							
Al_2_O_3_	SiO_2_	K_2_O	Fe_2_O_3_	TiO_2_	MgO	CaO	others
41.90	52.90	0.77	1.60	1.80	0.19	0.17	0.67
**Sodium Silicate Solution**							
SiO_2_	Na_2_O	H_2_O					
27.40	8.15	64.45					

**Table 2:** Composition (wt %) of the samples used in this study.

Mix ID	MK	FA	SS	NaOH	NaOH _soln_	Resin
G-MK	41.6	-	50.0	8.4	-	-
G-MK-Ep10	37.4	-	45.0	7.6	-	10
G-MK-Ep20	33.3	-	40.0	6.7	-	20
G-FA	-	60.2	19.9	-	19.9	-
G-FA-Ep10		54.2	17.9	-	17.9	10
G-FA-Ep20		48.2	15.9	-	15.9	20

MK = metakaolin; FA = fly ash; SS = sodium silicate solution; NaOH _soln_ = aqueous sodium hydroxide solution 10 M; Resin = Epojet^®^ Mapei S.p.A. [34].

**Table 3:** Thermal properties of the neat geopolymer (G-FA), pure epoxy resins (Epojet), and the composite specimen (G-FA-Ep20).

Mix ID	Weight Loss Starting Temperature (°C)	Weight Loss Ending Temperature (°C)	Weight Loss at 200 °C (wt %)	Weight Loss at 400°C (wt %)	Residual at 800°C (wt %)
G-FA	30	750	7.2	8.7	85
Epojet	250	650	2.1	39.4	5
G-FA-Ep20	30	700	2.6	9.2	78
